# Finite Element Analysis in Polymer-Based Adhesive Dental Restorations: A Narrative Review on Material Behavior, Methodological Validity, and Clinical Relevance

**DOI:** 10.3390/polym18050580

**Published:** 2026-02-27

**Authors:** Angelo Aliberti, Mario Caggiano, Mirko Piscopo, Roberta Gasparro, Mariangela Cernera, Niccoló Giuseppe Armogida, Pietro Ausiello

**Affiliations:** 1 Department of Neuroscience, Reproductive Science and Dentistry, University of Naples Federico II, 80131 Naples, Italy; ange.aliberti@studenti.unina.it (A.A.); mirk.piscopo@studenti.unina.it (M.P.); roberta.gasparro@unina.it (R.G.); mariangela.cernera@unina.it (M.C.); niccologiuseppe.armogida@unina.it (N.G.A.); 2 Department of Medicine, Surgery and Dentistry, Scuola Medica Salernitana, University of Salerno, 84081 Baronissi, Italy; macaggiano@unisa.it

**Keywords:** finite element analysis, adhesive restorative dentistry, polymer-based dental materials, direct composite restorations, indirect adhesive restorations, adhesive polymers, polymerization shrinkage, stress distribution, cuspal deflection

## Abstract

Finite element analysis (FEA) is increasingly used in conservative and restorative dentistry to investigate the mechanical behavior of adhesive direct and indirect polymer-based restorations. Despite the growing number of FEA-based studies, the literature currently lacks a dedicated critical synthesis specifically addressing the methodological validity and clinical interpretability of FEA in adhesive restorative dentistry. This narrative review critically examines the current literature on the application of FEA in adhesive restorative dentistry, with particular attention to class I to class V cavities in anterior and posterior teeth restored with direct or indirect polymeric materials, including inlays, onlays, overlays, and tabletop restorations. A structured, non-systematic search of major databases was conducted, and selected studies were qualitatively appraised with emphasis on modeling assumptions, stress distribution, and clinical meaning. Unlike previous broad overviews of dental biomechanics, this review provides a clinically oriented framework for interpreting FEA findings across restorative strategies. FEA consistently identifies trends related to cavity configuration, cuspal support, restoration design, material stiffness, polymerization shrinkage, and adhesive interface behavior, helping to explain clinically observed failure patterns and supporting minimally invasive approaches. However, simplified material models, idealized bonding conditions, and static loading protocols limit prediction of long-term performance. When interpreted within these constraints and integrated with experimental and clinical evidence, FEA remains a valuable complementary tool for rational restorative decision-making.

## 1. Introduction

Contemporary conservative and restorative dentistry is deeply grounded in the principles of tissue preservation, adhesion, and biomechanical integrity. Over the last decades, the transition from mechanically retained restorations toward adhesive strategies has profoundly reshaped clinical decision-making, promoting minimally invasive approaches aimed at preserving sound tooth structure while restoring function and esthetics [1,2,3]. This paradigm shift has been driven by continuous improvements in adhesive polymeric systems, including resin-based direct composites and partial indirect restorations, which have expanded the range of treatment options available for managing structural tooth loss across all cavity classes [4]. Direct adhesive restorations remain the first-line treatment for many clinical situations involving class I and II posterior lesions, as well as class III, IV, and V defects, owing to their conservative nature, reparability, and favorable cost–benefit ratio [5,6]. Nevertheless, as cavity dimensions increase and key structural components such as marginal ridges or cuspal support are compromised, particularly in extensive class II restorations, the mechanical behavior of the restored tooth changes substantially [7,8]. Loss of structural continuity results in increased cuspal deflection and altered stress distribution within enamel and dentin, potentially predisposing the tooth–restoration complex to fatigue-related failure over time [9,10]. In these situations, indirect polymeric adhesive restorations, including inlays, onlays, overlays, and more recently tabletop dental restorations, have gained increasing acceptance as conservative alternatives to full-coverage crowns, providing cuspal reinforcement while limiting unnecessary removal of sound tooth structure [11,12].

Although posterior teeth have traditionally received the greatest attention in biomechanical investigations [13], anterior adhesive restorations represent an equally relevant domain within conservative dentistry [14]. Class III and IV resin-based restorations are exposed to complex oblique and tensile loading patterns during function and parafunction [15], with stress concentration frequently occurring at the incisal edge and along the polymeric adhesive interface. These mechanical characteristics render anterior teeth especially sensitive to restoration design, material stiffness, and bonding strategy, while cervical class V lesions are further influenced by tooth flexure and non-axial loading [16].

The success of both direct and indirect adhesive restorations is inherently linked to biomechanical factors [17]. Occlusal loads are transmitted through a complex assembly consisting of differently stiff (E) materials such as enamel (80 GPa), dentin (20 GPa), adhesive layers (1 GPa), polymeric-based restorative materials (12 to 25 GPa), and, in the case of indirect restorations, luting agents (6 GPa) [18]. Cavity configuration, remaining tooth structure, restorative design, and material properties interact in a non-linear manner, influencing stress distribution and deformation patterns under functional loading [19]. While in vitro studies provide valuable information on fracture resistance, marginal integrity, and fatigue behavior, they are inherently limited by specimen variability and by their inability to directly visualize internal stress and strain distributions [20,21].

Within this complex biomechanical framework, Finite Element Analysis (FEA) has progressively emerged as a widely adopted numerical method in conservative and restorative dentistry. By discretizing complex geometries into finite elements and assigning material properties and boundary conditions, FEA enables the simulation of stress and deformation fields within tooth–restoration complexes that are otherwise inaccessible through experimental approaches alone [22]. Since its early applications in dental biomechanics, FEA has been increasingly used to investigate cuspal deflection in large class I and II dental polymeric-based restorations, stress development associated with polymerization shrinkage, cervical stress concentration in class V lesions, and stress distribution in indirect adhesive resin-based restorations with different materials and designs [23,24,25]. In anterior teeth, FEA has been applied to analyze class IV dental polymeric-based restorations, the mechanical impact of incisal edge involvement, and the stress response of cervical lesions under oblique loading [26]. Although these applications are less frequently represented in the literature compared with posterior restorations, available numerical studies have provided valuable insights into the role of tensile stresses, tooth flexure, and adhesive interface behavior in anterior restorative scenarios [27,28]. The relative scarcity of FEA studies focusing on anterior teeth does not reflect limited clinical relevance but rather highlights an imbalance in the current literature that warrants critical consideration when interpreting numerical evidence.

Because of its versatility and accessibility, FEA-based studies have become increasingly prevalent in the restorative literature and are often cited to support clinical concepts such as cuspal coverage, polymeric restorative material selection, thickness optimization, and cavity design [29]. However, despite its widespread use, the methodological validity and clinical meaning of FEA outcomes remain insufficiently discussed. Finite element models inevitably rely on simplifying assumptions to ensure computational feasibility. Linear elastic behavior is frequently assigned to materials known to exhibit viscoelastic or anisotropic properties, adhesive interfaces are commonly modeled as perfectly bonded, and occlusal loading is often simplified to static forces applied at idealized contact points [30]. While these assumptions may be acceptable for comparative analyses, they raise important concerns regarding the biological and clinical realism of numerical predictions. Furthermore, interpretation of FEA results in restorative dentistry often relies on qualitative color-coded stress maps and peak stress values. Although visually intuitive, such outputs are not always associated with validated failure criteria or clinically meaningful endpoints, increasing the risk of overinterpretation [29,31]. This issue is particularly critical in adhesive dentistry, where restoration failure is rarely dictated by single-load catastrophic fracture, but more commonly by cumulative fatigue damage, marginal degradation, and deterioration of the adhesive interface over time [32].

Notably, despite the extensive and growing number of FEA-based studies in adhesive direct and indirect restorative dentistry, the literature currently lacks narrative or systematic reviews specifically dedicated to critically evaluating the validity and effectiveness of FEA in this field. Previous studies have either addressed FEA within broader dental biomechanics contexts or have focused on specific clinical techniques, without providing a comprehensive, clinically oriented discussion on how numerical findings should be interpreted and integrated into adhesive restorative decision-making across different cavity classes and tooth groups [33].

Considering the growing use of FEA to guide restorative design and material selection, a focused evaluation of its methodological assumptions and clinical interpretability within adhesive restorative dentistry appears warranted. Therefore, the aim of this review is to critically analyze the current evidence on the application of Finite Element Analysis in conservative and restorative dentistry, with particular emphasis on adhesive resin-based direct and indirect restorations in class I to class V cavities involving both posterior and anterior teeth. Adopting an intentionally narrative and clinically oriented perspective, this review goes beyond a descriptive overview of numerical studies to evaluate the methodological assumptions underlying FEA models, discuss their validity and efficiency as investigative tools, and examine the extent to which numerical findings can be meaningfully translated into clinical practice. By focusing on interpretative and methodological aspects rather than on quantitative synthesis, this review aims to clarify the role of FEA in contemporary adhesive restorative dentistry and to provide a rational framework for its appropriate interpretation and use in both research and clinical settings.

The manuscript is structured into sections addressing the application of FEA in direct and indirect adhesive restorations, followed by a discussion on methodological considerations, clinical implications, limitations and future perspectives.

## 2. Materials and Methods

### 2.1. Literature Search Strategy

This narrative review was designed to provide a critical and clinically oriented synthesis of the existing literature on the application of finite element analysis (FEA) in conservative and restorative dentistry, with specific emphasis on adhesive resin-based direct and indirect restorations. Given the intrinsic heterogeneity of numerical modeling strategies, restorative designs, and outcome variables, a narrative review framework was considered the most appropriate methodological approach to explore issues of methodological validity, interpretative limits, and clinical relevance rather than to perform a quantitative synthesis.

A structured, non-systematic literature search was conducted using the PubMed/MEDLINE, Scopus, and Web of Science databases. The search included articles published from January 2000 to December 2025 and was restricted to English-language publications. This time frame was selected to encompass the period during which FEA progressively became established as a commonly adopted investigative tool in dental biomechanics and adhesive restorative research.

Search terms were combined using Boolean operators and included: “finite element analysis”, “finite element method”, “adhesive dentistry”, “restorative dentistry”, “direct restoration”, “composite restoration”, “indirect restoration”, “adhesive restoration”, “inlay”, “onlay”, “overlay”, “tabletop restoration”, “partial coverage restoration”, “anterior restoration”, “posterior restoration”, “polymeric dental materials”, “resin-based composites”, “polymer-based restorations”, “class I cavity”, “class II cavity”, “class III cavity”, “class IV cavity”, “class V cavity”, “cuspal deflection”, “polymerization shrinkage”, “interfacial stress”, “stress distribution”, “primary teeth restorations”, “pediatric dentistry”, “permanent teeth”, and “dental biomechanics”.

To ensure comprehensive coverage of the literature, a supplementary manual search was undertaken. This involved a systematic screening of the reference lists from all pertinent articles identified in the initial electronic search. Additionally, targeted manual searches were conducted within key specialty journals in the field, including Dental Materials, Journal of Dentistry, The Journal of Prosthetic Dentistry, and Clinical Oral Investigations. This combined electronic and manual strategy was implemented to identify seminal, high-impact, or complementary studies that may not have been captured through database searches alone, thereby enhancing the breadth and robustness of the narrative synthesis.

### 2.2. Eligibility Criteria and Study Selection

Eligible publications included original finite element studies, in vitro investigations incorporating numerical modeling, narrative and systematic reviews, communications and methodological papers addressing the biomechanical behavior of adhesive resin-based direct and indirect restorations in both primary and permanent teeth, in either vital or endodontically treated dentitions, provided that the restorative approach was based on adhesive principles and partial-coverage designs. Both posterior and anterior teeth were considered, and studies evaluating class I to class V cavities restored with adhesive techniques were included. Study selection was guided by relevance to conservative and restorative dentistry and by the potential contribution of numerical findings to clinical interpretation.

Studies were excluded if they focused on finite element analysis of endodontic post systems, including fiber posts and other intraradicular posts, or on endocrowns, as these restorations involve distinct biomechanical principles and failure mechanisms beyond the scope of this review. In addition, studies investigating FEA of prosthetic restorations, such as full-coverage crowns, fixed dental prostheses and removable partial dentures, were excluded. Also, FEA investigations about overdentures, or implant-supported restorations, including full-arch rehabilitations such as all-on-four concepts, were excluded. In addition, studies addressing maxillary or mandibular bone reconstructions, implant-related biomechanics, orthodontic biomechanics, endodontic instrumentation, or maxillofacial structures unrelated to conservative restorative procedures were not taken into consideration. Furthermore, studies focusing on finite element analysis of ceramic veneers in anterior teeth were excluded, as veneer restorations represent a distinct prosthetic-restorative category with different biomechanical and clinical considerations. Purely engineering-oriented studies lacking clear dental or clinical applicability were not considered, nor were papers with insufficient methodological detail to allow meaningful assessment of model assumptions, boundary conditions, or outcome interpretation.

These exclusions were defined a priori to preserve conceptual and biomechanical homogeneity within the scope of adhesive, partial-coverage, polymer-based restorative strategies and to avoid conflating restorative approaches characterized by fundamentally different retention principles, load transfer mechanisms, and failure patterns.

Following the initial database search, titles and abstracts were screened for thematic relevance to adhesive restorative dentistry. Full texts were then examined to assess their methodological and clinical contribution to the scope of this narrative review. Studies that did not align with the predefined thematic scope of adhesive restorative dentistry or that lacked sufficient methodological detail were excluded after full-text assessment. In accordance with the narrative and concept-driven design of this review, no predefined quantitative inclusion thresholds or formal risk-of-bias assessment tools were applied. Given the marked heterogeneity in model construction, material property assignment, loading conditions, and reported outcome parameters across FEA studies, a quantitative aggregation of results was not considered methodologically appropriate. Instead, emphasis was placed on qualitative appraisal of the selected studies, focusing on key modeling aspects such as geometric representation, material property assignment, simulation of adhesive interfaces, loading and boundary conditions, and validation against experimental or clinical data when available. Attention was given to how numerical outcomes were interpreted and whether conclusions were framed in a clinically meaningful and methodologically transparent manner.

Because geometric simplifications, homogeneous material modeling, and idealized bonding conditions are common features of most dental FEA studies, they were not treated as exclusion criteria. Their methodological implications are instead examined in the specific section devoted to model validity and interpretative limits. The included literature was analyzed and synthesized thematically rather than chronologically or quantitatively. Studies were grouped according to restorative approach (direct versus indirect), cavity class, tooth group (posterior versus anterior), and dentition (primary versus permanent), as well as by primary biomechanical focus (e.g., cuspal deflection, polymerization shrinkage, interfacial stress, stress distribution within dental tissues or restorative materials). This thematic organization was intended to facilitate a critical discussion of recurring modeling assumptions, methodological strengths and limitations, and the translational relevance of FEA findings in contemporary adhesive restorative dentistry.

## 3. Results

The results of the present narrative review highlight the extensive and heterogeneous use of finite element analysis (FEA) in adhesive restorative dentistry. Across the retrieved literature, FEA has been applied to a wide range of restorative scenarios, tooth types, and cavity designs, reflecting the growing interest in understanding the mechanical behavior of adhesive polymeric-based dental restorations under controlled numerical conditions. Rather than yielding uniform or directly comparable outcomes, the available studies provide a spectrum of mechanical insights that are highly dependent on cavity configuration, restorative strategy, and modeling assumptions.

For this reason, the results were not synthesized in a quantitative or study-by-study manner but instead organized into clinically meaningful macro-areas. The literature was first distinguished according to direct versus indirect resin-based adhesive restorations, and subsequently categorized by cavity class, restorative design, and tooth group (anterior versus posterior). This approach was adopted to reflect real-world restorative decision-making and to facilitate interpretation of numerical findings within a clinical framework.

Within each macroarea, FEA findings consistently focus on stress distribution, cuspal deflection, interfacial stress development, and deformation patterns of the tooth–restoration complex. However, the degree to which these outcomes can be translated into clinical implications varies considerably, depending on the extent of cavity preparation, the presence or absence of cuspal coverage, and the simplifications inherent to the numerical models. The following subsections therefore present the results thematically, emphasizing recurring mechanical trends and clinically relevant patterns rather than isolated numerical values.

### 3.1. Finite Element Analysis in Direct Adhesive Restorations

#### 3.1.1. Class I Direct Adhesive Restorations

Finite element analysis (FEA) has been extensively employed to investigate the mechanical behavior of class I direct adhesive polymer-based restorations, which represent a relatively conservative cavity configuration characterized by a high configuration factor (C-factor) and limited freedom for deformation. Owing to these characteristics, class I cavities have frequently been adopted as reference numerical models to explore fundamental mechanical interactions between polymerization shrinkage, restorative material properties, adhesive interface behavior, and occlusal loading conditions. Early three-dimensional FEA investigations primarily focused on polymerization shrinkage–induced stresses developing within bonded class I restorations. Rodrigues et al. demonstrated that an increase in cavity C-factor does not necessarily result in a proportional increase in interfacial stress peaks, challenging the traditional assumption that cavity configuration alone dictates shrinkage stress magnitude [34]. Their numerical findings indicated that stress distribution patterns are strongly influenced by cavity geometry, remaining wall thickness, and boundary conditions, with stress concentrations variably localized along axial walls and at the pulpal floor.

Subsequent numerical studies further explored the influence of polymeric restorative material chemistry on shrinkage stress development. Comparative FEA models evaluating methacrylate-based composites and alternative low-shrinkage resin systems, such as silorane-based materials, consistently reported reduced polymerization shrinkage and lower stress concentration at the tooth–restoration interface when non-methacrylate chemistries were employed [35]. These numerical observations provided mechanical support for the clinical rationale underlying the development of low-shrinkage composite formulations for posterior direct restorations. More recent CAD–FE investigations expanded the analysis of class I cavities to include combined polymerization shrinkage and occlusal loading scenarios. Ausiello et al. evaluated molar class I restorations restored with bulk-fill resin composites and alkasite materials, demonstrating that stress concentration under simulated masticatory loads was predominantly localized at the enamel–restoration interface [36]. Notably, the introduction of polymeric or ionic base materials beneath bulk restorations did not consistently result in a reduction in stress magnitude within enamel and dentin, suggesting that load transfer mechanisms and material stiffness may play a more decisive role than the mere presence of an intermediate layer.

The mechanical implications of multilayer restorative strategies in class I cavities have also been investigated through FEA. Several studies employing bi-layer restorative approaches combining resin composites with glass ionomer cements reported reduced stress transmission toward dentin and the pulpal floor compared with single-layer bulk composite restorations [37,38]. These numerical findings support the hypothesis that intermediate layers with lower elastic modulus and reduced polymerization shrinkage may partially mitigate stress concentration in deep class I cavities, with lower stress–strain deformation at the dentin–adhesive interface.

More recently, FEA studies have addressed the mechanical behavior of bioactive restorative resin-based materials in class I direct restorations. Numerical analyses comparing conventional resin composites with hybrid glass ionomer-based or bioactive restorative materials demonstrated stress distribution patterns closer to those observed in sound teeth, especially under combined shrinkage and occlusal loading conditions [39,40]. These findings suggest that material chemistry and elastic behavior significantly influence stress redistribution within the tooth–restoration complex, potentially affecting the long-term biomechanical stability of class I adhesive restorations.

#### 3.1.2. Class II Direct Adhesive Restorations and Mesio-Occlusal-Distal (MOD) Restorations

Building on the mechanical framework established by finite element analyses of class I direct adhesive resin-based restorations, the application of FEA to class II mesio-occlusal-distal (MOD) restorations has received substantially greater attention in the literature. This reflects the markedly different mechanical scenario associated with MOD cavity preparation, which involves the loss of both marginal ridges and results in a pronounced reduction in structural stiffness. Consequently, MOD restorations represent the most mechanically demanding configuration among direct adhesive resin-based restorations and are frequently adopted as reference models for investigating stress amplification and cuspal deformation.

Across the included studies, FEA consistently demonstrates that MOD cavity preparation leads to a significant increase in cuspal deflection under occlusal loading compared with intact teeth, class I cavities, and class II restorations involving the loss of a single marginal ridge [41,42,43,44]. Stress concentration patterns are characterized by elevated tensile stresses at the cervical enamel, internal line angles, and along the dentin–adhesive interface, particularly in proximity to the gingival margins of the proximal boxes. These effects are systematically exacerbated under non-axial or oblique loading conditions, which more closely simulate functional and parafunctional contacts, resulting in higher stress gradients and deformation levels than those observed in class I restorations [45,46].

A substantial portion of the FEA literature on MOD restorations has focused on the interaction between polymerization shrinkage and functional loading. Numerical simulations indicate that shrinkage-induced stresses develop early during composite polymerization and subsequently superimpose on occlusal stresses during simulated mastication, generating complex and highly localized stress fields within enamel, dentin, and at adhesive interfaces [47,48]. Although absolute stress magnitudes vary considerably among studies due to differences in material models and boundary conditions, there is consistent agreement that MOD cavities are particularly susceptible to interfacial stress accumulation and stress amplification within the remaining tooth structure. Restorative polymeric material properties represent another recurrent variable in MOD finite element models. Comparative analyses consistently show that materials with higher elastic modulus promote greater stress transmission to enamel and dentin, leading to increased tensile stresses at cervical margins and internal line angles. Conversely, resin-based restorative materials with lower stiffness partially attenuate stress transfer by accommodating greater deformation, albeit with increased cuspal displacement [49,50]. Studies evaluating bulk-fill resin composites in MOD cavities report stress distributions broadly comparable to those of conventional incremental composites; however, variations in cuspal deflection, interfacial stress, and residual shrinkage stress have been observed depending on material formulation, curing assumptions, and viscoelastic behavior [51,52].

Several FEA investigations have specifically addressed the influence of cavity geometry and remaining tooth structure on the mechanical response of MOD restorations. Numerical evidence consistently indicates that increasing cavity width, reducing residual cusp thickness, and enlarging the isthmus region result in disproportionate increases in cuspal deflection and tensile stress concentration [53,54]. In this context, optimization-based finite element studies have explored alternative cavity geometries and internal line angle modifications, demonstrating that changes in restoration shape can significantly influence stress distribution and debonding behavior [55,56,57]. More recent FEA studies have expanded MOD analyses to include additional mechanical variables, such as thermomechanical loading and material property mismatches between restorative resin-based composites and dental tissues. These investigations highlight the restoration–enamel junction as a critical site for principal stress concentration under combined thermal and mechanical stimuli, further emphasizing the mechanical vulnerability of MOD restorations under clinically relevant conditions [58,59]. Moreover, numerical models evaluating reinforcement strategies in extensively prepared MOD cavities, including non-invasive structural reconnection approaches, demonstrate reduced cuspal deflection and stress concentration when macromechanical continuity between residual walls is restored [52,60].

#### 3.1.3. Class III Direct Adhesive Restorations

Compared with posterior class I and class II cavities, the application of finite element analysis to class III direct adhesive restorations remains markedly limited in the available literature. Despite the high clinical relevance of these restorations, particularly due to their location in the anterior esthetic zone and their exposure to complex functional and parafunctional loading patterns, only a small number of numerical investigations have specifically addressed their mechanical behavior. This scarcity of evidence contrasts sharply with the extensive FEA-based body of research available for posterior teeth and highlights a clear gap in contemporary restorative biomechanics.

The limited numerical evidence currently available suggests that class III cavities exhibit distinct mechanical characteristics when compared with posterior restorations [61]. Due to the reduced cavity volume and the predominance of enamel at the cavosurface margins, stress distribution patterns are strongly influenced by polymerization shrinkage rather than by occlusal loading alone [62]. In this context, three-dimensional FEA has been primarily employed to investigate shrinkage-induced stresses at the internal and marginal adhesive interfaces, rather than global tooth deformation or fracture-related outcomes.

The most recent and comprehensive contribution to this topic is a three-dimensional FEA study evaluating internal and marginal polymerization shrinkage stress in class III adhesive restorations restored using different resin composite combinations and layering strategies [27]. In that investigation, a detailed anatomical model of a maxillary central incisor with a distal class III cavity was developed, allowing for high-resolution assessment of stress distribution at both enamel and dentin interfaces. Polymerization shrinkage was simulated through thermal analogy, and stress patterns were analyzed using the Maximum Principal Stress criterion, which is commonly adopted in adhesive dentistry FEA studies to identify tensile stress concentrations.

The numerical results demonstrated that marginal stress concentrations were predominantly localized at the enamel cavosurface bevel, regardless of the restorative technique employed. Peak stress values at the enamel interface were consistently higher than those observed in dentin, reflecting the pronounced elastic modulus mismatch between enamel and resin composite materials. In contrast, internal dentin stress was more sensitive to the restorative strategy, particularly to the use of low-modulus flowable bulk-fill liners combined with conventional composite increments. Configurations incorporating a flowable base exhibited higher internal dentin stress value, suggesting a potential mechanical vulnerability of the adhesive interface under shrinkage-dominated conditions.

Interestingly, despite variations in layering technique and composite type, the overall marginal stress distribution patterns among the simulated class III restorations were relatively homogeneous. This finding suggests that, within the limitations of static FEA models, the influence of restorative technique on marginal stress concentration in class III cavities may be less pronounced than in more extensive posterior restorations, such as class II MOD cavities [63]. However, the consistently elevated stress levels at the enamel bevel region underline the mechanical importance of marginal design and enamel bonding in anterior adhesive restorations. From a broader perspective, the paucity of FEA studies on class III restorations limits the ability to draw generalized conclusions regarding optimal restorative strategies, material selection, or cavity design for anterior teeth. Most available numerical evidence remains exploratory and technique-oriented, focusing predominantly on polymerization shrinkage rather than on combined mechanical, thermal, and fatigue loading conditions. Consequently, current FEA findings for class III restorations should be interpreted as hypothesis-generating rather than definitive.

#### 3.1.4. Class IV Direct Adhesive Restorations

Within the finite element analysis literature on adhesive restorative dentistry, class IV direct polymeric-based dental restorations have been only marginally investigated when compared with posterior cavity designs. This limited scientific attention is particularly striking given the high clinical relevance of class IV restorations, which are frequently indicated for the management of traumatic incisal defects and extensive anterior tooth fractures. Despite their functional and esthetic importance, the mechanical behavior of class IV polymeric-based restorations has received comparatively little attention in FEA-based research.

From a mechanical perspective, class IV restorations differ substantially from posterior cavities due to the involvement of the incisal edge and the predominance of tensile and shear stresses under functional loading [64]. Anterior teeth are subjected to complex oblique loading patterns during protrusive and lateral mandibular movements, making cavity geometry, marginal configuration, and stress transfer at the adhesive interface critical factors influencing restoration performance [65,66,67,68].

One of the few dedicated three-dimensional FEA studies in this area, conducted by Xu et al. [26], evaluated the influence of cavity design on stress distribution and fracture behavior in maxillary central incisors restored with direct composite resin. Anatomically detailed models were used to simulate class IV cavities with different marginal designs, including bevels, chamfers, stair-step chamfers, and butt joint preparations, under clinically relevant loading conditions. The numerical results demonstrated that cavity design significantly affected stress distribution within both the restorative material and surrounding dental tissues [26]. Preparations incorporating chamfered or stair-step margins exhibited more homogeneous stress patterns, whereas bevel and butt joint designs were associated with higher stress concentration, particularly under oblique loading [26].

In addition, the FEA findings were supported by complementary fracture resistance testing, which provided qualitative validation of the numerical outcomes. Cavity designs associated with more favorable stress distribution patterns also showed improved fracture resistance and more favorable failure modes, characterized by a higher prevalence of cohesive fractures rather than adhesive failures at the restoration margins. Overall, the limited FEA evidence available suggests that cavity design plays a decisive role in governing the mechanical behavior of class IV resin-based direct adhesive restorations. However, the scarcity of dedicated numerical studies limits the ability to draw generalized conclusions for anterior adhesive restorations. Further high-quality FEA investigations incorporating advanced loading protocols and refined material modeling are required to achieve a mechanical understanding comparable to that currently available for posterior adhesive restorations.

#### 3.1.5. Class V Direct Adhesive Restorations

Class V direct resin-based adhesive restorations represent a distinct mechanical scenario within conservative dentistry, as they are typically located in the cervical region of the tooth, an area characterized by complex stress patterns driven by non-axial occlusal loading, tooth flexure, and pronounced material–tooth interfacial interactions. Unlike occlusal or proximal restorations, class V cavities are positioned close to the cemento–enamel junction, where reduced enamel thickness, dentin exposure, and the transition between tissues with markedly different elastic properties contribute to stress amplification under functional loading conditions [69,70,71].

Finite element analysis has been widely applied to investigate the mechanical behavior of class V adhesive resin-based dental restorations, particularly in the context of non-carious cervical lesions and wedge-shaped defects. Early three-dimensional FEA studies demonstrated that the creation of a cervical cavity introduces a structural discontinuity that alters the stress field within the tooth, with peak tensile stresses consistently localized at the gingival margin and along the adhesive interface [72]. These numerical findings provided a mechanical explanation for the clinically observed susceptibility of class V restorations to marginal degradation, debonding, and reduced long-term retention.

Material-related variables have emerged as critical determinants of stress modulation in class V restorations. Multiple FEA investigations have reported that restorative materials with higher elastic modulus tend to transmit stresses more directly to the surrounding dental tissues, resulting in increased stress concentration at the cervical margins. In contrast, materials with lower stiffness exhibit greater deformation and stress absorption capacity, which may reduce peak interfacial stresses while increasing restorative strain [73,74]. Comparative numerical analyses involving resin composites, glass ionomer cements, and hybrid or bioactive materials consistently highlight the importance of elastic compatibility between restorative materials and cervical dentin [75,76,77]. Loading direction has been repeatedly identified as one of the most influential parameters affecting the mechanical performance of class V restorations. Finite element simulations demonstrate that oblique or eccentric occlusal loading generates substantially higher tensile and shear stresses in the cervical region than purely axial loading, thereby intensifying stress concentration at the tooth–restoration interface [70,78]. This mechanical behavior aligns with clinical observations linking occlusal interferences, parafunctional habits, and eccentric loading patterns to the development and progression of cervical lesions.

Cavity geometry and preparation design have also been systematically investigated through FEA. Numerical models comparing different class V cavity shapes revealed that sharp internal angles and wedge-shaped configurations are associated with higher stress concentration, whereas smoother, rounded cavity designs tend to distribute stresses more evenly along the adhesive interface and surrounding dentin [74,79]. These findings support minimally invasive preparation concepts that aim to reduce stress amplification while preserving cervical tooth structure. Thermo-mechanical effects have further expanded the scope of finite element investigations in class V polymeric-based restorations. Studies incorporating thermal loading have shown that temperature fluctuations induce additional stresses at the adhesive interface due to mismatched coefficients of thermal expansion between restorative materials and dental tissues [75]. When combined with mechanical loading, thermal stresses have been shown to exacerbate interfacial stress peaks, potentially contributing to marginal breakdown and restoration failure over time [80].

More recent FEA studies have incorporated advanced modeling strategies, including refined mesh convergence, simulation of periodontal ligament support, and multi-material interface representation, to enhance the physiological relevance of numerical outcomes [81,82]. These investigations emphasize that realistic boundary conditions and accurate material property assignment are essential for meaningful mechanical interpretation, as oversimplified assumptions may lead to misleading stress predictions.

Finally, the finite element literature indicates that class V direct resin-based adhesive restorations are governed by a multifactorial mechanical environment in which material properties, cavity design, loading direction, and environmental factors interact in a complex manner. While FEA does not directly predict clinical longevity, it provides valuable insight into stress concentration patterns and failure-prone regions that are difficult to assess experimentally. Within this context, class V restorations remain one of the most mechanically sensitive applications of direct adhesive dentistry.

### 3.2. Finite Element Analysis in Indirect Adhesive Restorations

Within the category of indirect polymeric-based restorations, finite element analysis studies can be further stratified into intracoronal inlays, partial cuspal coverage restorations such as onlays and overlays, and ultra-conservative resin-based occlusal veneers or tabletop adhesive restorations. Although these restorative approaches differ in extension and geometry, they share a common mechanical foundation based on adhesive retention and stress transfer through bonded interfaces rather than macromechanical preparation features [83,84,85,86]. Finite element analysis has been widely adopted to investigate indirect adhesive resin-based restorations as conservative alternatives to full-coverage solutions, with the specific goal of understanding how bonded restorations restore stiffness and redistribute functional loads in structurally compromised teeth [85,87]. Early numerical studies on intracoronal restorations consistently showed that adhesively luted inlays can partially recover the stiffness of teeth weakened by extensive cavity preparations, reducing stress concentration within residual dentin when compared with unrestored cavities; nevertheless, stress peaks frequently persist at internal line angles and in cervical enamel regions, especially in class II and MOD-type geometries [83,84,85,87,88,89]. This pattern has been repeatedly confirmed in subsequent tooth-specific simulations, supporting the concept that the mechanical response of indirect polymeric-based adhesive restorations is governed not only by the restorative material but also by cavity configuration and residual tooth structure [90].

A central theme across the FEA literature is the influence of restorative material properties on stress distribution within the tooth–restoration complex. Comparative numerical analyses indicate that higher-modulus restorative materials (E), commonly ceramic, tend to concentrate stresses within the restoration and at the cement interface, whereas resin-based or more compliant materials distribute stresses more gradually into enamel and dentin [83,84,85,86,87,91,92]. Importantly, from an adhesive dentistry perspective, these effects are consistently interpreted in relation to the integrity of bonded interfaces and the mechanical compatibility between restoration, resin cement, and tooth substrate, rather than in purely “prosthetic” terms [82,83,88,93]. Studies that explicitly examine the adhesive layer further support the concept that the mechanical behavior of the interfacial region can influence how stresses are transferred between restoration and tooth, and, therefore, how stress concentrations localize at critical enamel margins or dentin interfaces [82,94,95].

Restoration thickness and layer configuration represent additional variables extensively investigated through FEA. Numerical models evaluating indirect restorations consistently suggest that increasing restoration thickness can reduce tensile stress peaks at the adhesive interface and within the polymeric restorative material, particularly for cuspal coverage designs, whereas thin restorations are more prone to stress concentration under oblique loading conditions [96,97]. At the same time, simulations evaluating intermediate resin layers indicate that compliant bases/liners may modify stress transfer: in some models, lower-modulus intermediate layers reduce stresses in dentin but increase tensile stresses within brittle restorative materials, underscoring that “stress buffering” is not uniformly beneficial and must be interpreted within the full multilayer adhesive complex [86,87,98]. These results reinforce the need to consider restoration thickness and interfacial stratification (restorative material–cement–tooth) as a coupled system rather than optimizing any single layer in isolation [82,85,99].

Polymerization shrinkage of resin-based luting agents is a particularly relevant issue for indirect polymeric-based adhesive restorations because, while indirect techniques reduce the volume of polymerizing resin compared with direct restorations, shrinkage-related stresses may still accumulate at bonded interfaces where cement is constrained by cavity walls and by the restoration’s internal surface [100,101,102]. Finite element studies explicitly simulating cement polymerization show that shrinkage-induced stresses develop primarily within the cement/adhesive layer and at the bonded interfaces and may superimpose on functional occlusal stresses during subsequent loading [103]. The magnitude and distribution of shrinkage stresses are influenced by restoration geometry, cement thickness, and elastic mismatch among restoration, cement, and dental tissues, making polymerization stress a persistent mechanical variable in indirect adhesive dentistry rather than a negligible phenomenon [104].

Onlays and overlays, treated in the conservative-restorative sense as adhesively bonded cuspal coverage restorations, have been examined through FEA to evaluate their ability to reduce cuspal deflection and shift stress concentrations away from weakened residual walls [105,106,107]. Numerical models generally indicate that cuspal coverage can reduce deformation of remaining cusps when compared with intracoronal restorations, though stress redistribution is strongly dependent on coverage design, material stiffness, and the behavior of the adhesive/cement interface [83,84,85,86,89,91]. In this context, studies focusing on preparation design and ceramic thickness further emphasize how geometric variables interact with adhesive retention: design changes that increase structural support or thickness may reduce tensile stress peaks yet may also move stress concentration toward the restoration–cement interface depending on material properties and loading direction [108]. More recently, FEA has been applied to ultra-conservative resin-based occlusal veneers and tabletop restorations, where adhesive retention and enamel bonding become central determinants of mechanical viability because preparation is minimal and restorations may be thin [109]. Numerical investigations consistently localize high stresses at the enamel–cement interface and within the luting layer, particularly under non-axial or thermomechanical loading, highlighting that even small variations in thickness, margin configuration, and cement properties can meaningfully alter stress distribution in these restorations [83,84,85,86,87,88,91,95]. This body of work supports the conceptualization of resin-based tabletop restorations as “adhesive-driven” restorations where the integrity and mechanical behavior of the bonded interface is at least as relevant as the stiffness of the restorative material itself [108].

Methodological advances have further improved the translational relevance of FEA in indirect adhesive dentistry, including workflows based on detailed anatomical geometries, refined modeling of supporting structures, and more explicit attention to interfacial variables such as cement thickness and adhesive layer properties [110]. These approaches enable more realistic interpretation of stress pathways across enamel, dentin, cement, and restorative materials, and facilitate comparison between restorative designs under controlled boundary conditions [111].

To facilitate a structured and clinically oriented synthesis of the heterogeneous finite element literature reviewed, the main FEA-derived biomechanical insights, intrinsic methodological limitations, and related clinical implications across the different restorative scenarios are summarized in Table 1.

## 4. Discussion

### 4.1. Biomechanical Interpretation of FEA Findings in Adhesive Restorative Dentistry

The present narrative review underscores how finite element analysis has substantially advanced the biomechanical understanding of adhesive restorative dentistry, particularly in clinical scenarios where direct experimental assessment of internal stress and strain distribution is not feasible. Across both direct and indirect polymeric-based restorations, FEA consistently demonstrates that the mechanical behavior of the restored tooth is governed by a complex interaction between cavity geometry, residual tooth structure, restorative material properties, and the characteristics of the adhesive interface [23,83,84,87]. In posterior teeth, numerical evidence converges in showing that the loss of marginal ridges and cuspal support, typical of extensive class II and mesio-occlusal-distal restorations, results in pronounced alterations in stress distribution and cuspal deflection under functional loading [112]. Finite element simulations consistently localize tensile stress peaks at internal line angles, cervical enamel, and adhesive interfaces, providing a biomechanical explanation for the increased susceptibility of extensively restored posterior teeth to marginal degradation and fracture over time [70,83,113]. These findings help rationalize the clinical transition from intracoronal restorations toward cuspal coverage strategies when structural integrity is compromised.

In parallel, recent advancements in resin-based composite technology have introduced low-shrinkage monomer systems, bulk-fill formulations with modified polymerization kinetics, and bioactive or ion-releasing composites designed to modulate physicochemical behavior in the oral environment [27,39,40]. Although conventional FEA models primarily capture their immediate elastic response, these material developments highlight the evolving relationship between composite formulation and biomechanical performance, reinforcing the need for continuous updating of material parameters within numerical simulations.

For anterior teeth, FEA studies, although less numerous, highlight distinct biomechanical challenges related to non-axial loading, tensile stresses, and stress concentration at the incisal edge and along palatal or proximal adhesive interfaces [26,27]. In class III and IV dental restorations, numerical models indicate that restoration design, material stiffness, and bonding strategy critically influence stress transfer during function and parafunction, particularly under oblique loading conditions [26,27]. Similarly, class V restorations exhibit a unique biomechanical profile, with FEA consistently showing stress amplification at the cervical region due to tooth flexure, elastic mismatch between enamel and dentin, and non-axial occlusal forces [69,70,71,72,76].

A key contribution of FEA lies in its ability to differentiate the biomechanical behavior of direct versus indirect resin-based adhesive restorations under controlled conditions. While direct restorations are often associated with higher stress concentrations related to polymerization shrinkage and cavity configuration, indirect adhesive restorations generally exhibit more favorable stress redistribution under occlusal loading, particularly when cuspal coverage is provided [83,84,85,88,89]. However, numerical studies also demonstrate that indirect restorations remain highly sensitive to adhesive layer behavior, cement thickness, and material stiffness, reinforcing the concept that adhesive interfaces play a central biomechanical role regardless of the restorative approach [114]. Importantly, the convergence of FEA findings across diverse modeling strategies suggests that certain biomechanical trends are robust and clinically meaningful. These include the detrimental effect of extensive cavity geometry on stress distribution, the protective role of cuspal coverage in structurally compromised posterior teeth, and the critical influence of adhesive interfaces in both direct and indirect restorations [39,40,41,93,115]. When interpreted collectively rather than in isolation, these numerical insights provide a coherent biomechanical framework that supports contemporary principles of minimally invasive, adhesion-driven restorative dentistry.

### 4.2. Methodological Validity of FEA Models in Adhesive Restorative Dentistry

The methodological validity of finite element analysis (FEA) models represents a critical determinant of the reliability and clinical interpretability of numerical findings in adhesive restorative dentistry. Although FEA offers unparalleled control over geometric configuration, material behavior, and loading conditions, its outputs remain intrinsically dependent on the assumptions adopted during model construction, meshing strategy, constitutive modeling, and boundary condition definition [116]. In this context, stress metrics and failure criteria commonly used in dental FEA studies, such as maximum principal stress or von Mises stress, should be recognized as engineering constructs whose clinical meaning depends on model assumptions, validation procedures, and the mechanical characteristics of the simulated materials, as extensively discussed in the broader finite element literature [117].

One of the most frequent methodological simplifications concerns geometric representation. Many dental FEA investigations rely on idealized cavity preparations or averaged anatomical geometries, which may not fully capture the variability and structural complexity of clinical conditions [50,70,87,105]. While such simplifications facilitate mesh convergence and computational efficiency, studies based on micro-computed tomography reconstructions demonstrate that even subtle geometric variations, particularly at internal line angles and adhesive interfaces, can significantly influence stress localization. Geometric fidelity therefore emerges as a key factor in enhancing the biomechanical realism of numerical simulations.

Material property assignment constitutes another major source of variability across FEA studies in adhesive dentistry. Dental tissues and restorative materials are frequently modeled as homogeneous, isotropic, and linearly elastic, despite well-documented evidence of anisotropy, viscoelasticity, and time-dependent mechanical behavior, especially for dentin, resin-based composites, and adhesive layers [34,35,39]. Although linear elastic assumptions are often justified for comparative analyses under limited strain conditions, they restrict the ability of numerical models to reproduce clinically relevant phenomena such as fatigue damage accumulation, viscoelastic stress relaxation, and progressive interfacial degradation [33,118]. Consequently, FEA findings should be interpreted primarily as representations of short-term mechanical behavior rather than comprehensive predictors of long-term clinical performance.

The modeling of adhesive interfaces represents one of the most critical and methodologically sensitive aspects of FEA in adhesive restorative dentistry. In many investigations, interfaces between enamel, dentin, adhesive systems, resin cements, and restorative materials are assumed to be perfectly bonded, implying complete mechanical continuity without the possibility of damage initiation or debonding [119]. Although this assumption enhances numerical stability and allows standardized comparisons between restorative strategies, it does not reflect the heterogeneous and defect-sensitive nature of bonded interfaces observed clinically. The hybrid layer, incomplete resin infiltration, interfacial porosity, water sorption, and progressive chemical degradation introduce local mechanical discontinuities that cannot be reproduced under ideal bonding conditions. Finite element studies that explicitly model adhesive layers and resin cements as distinct entities demonstrate stress patterns that differ substantially from those obtained under simplified bonding assumptions, particularly when polymerization shrinkage is simulated [9,10]. These findings underscore that the adhesive interface should be regarded as an active mechanical component rather than a purely geometric boundary.

In this context, cohesive zone modeling approaches have been proposed as a more advanced and mechanistically consistent strategy for representing interfacial behavior within finite element simulations [120]. Cohesive zone models (CZM) are based on traction–separation relationships that define the mechanical response of the interface as a function of relative displacement between bonded surfaces, thereby allowing progressive stiffness degradation, damage initiation, and failure evolution to be simulated [121]. Unlike perfectly bonded models, which imply abrupt failure once a stress threshold is exceeded, cohesive formulations enable the representation of crack initiation and propagation according to energy-based fracture criteria derived from interfacial fracture mechanics principles [122]. Although their application within adhesive restorative dentistry remains relatively limited, cohesive interface approaches suggest that predicted stress redistribution patterns and failure initiation sites may differ substantially from those obtained under idealized bonding assumptions. In particular, cohesive formulations may better capture localized interfacial damage under tensile and shear loading, especially in extensive direct restorations and in thin indirect adhesive restorations where enamel margins and resin cement layers are mechanically critical. From a methodological standpoint, cohesive modeling does not eliminate the need for experimental validation of interfacial parameters; however, it provides a conceptual bridge between conventional stress-based FEA and fracture mechanics–oriented interpretations of adhesive failure, thereby enhancing the theoretical robustness of interfacial analysis [123].

Boundary conditions and loading protocols further influence the methodological validity of FEA outcomes. Static axial loading remains the most frequently adopted scenario, although it poorly reflects the multidirectional, cyclic, and time-dependent nature of physiological masticatory forces [20,21,22]. Numerical studies incorporating oblique loading, thermal variation, or transient thermo-mechanical conditions reveal substantially different stress distribution patterns, particularly in thin restorations and cervical regions, suggesting that simplified loading configurations may underestimate clinically relevant tensile and shear stress peaks [23,24,25,124]. The interpretative value of FEA findings is therefore closely linked to the extent to which loading conditions approximate functional reality.

Despite these limitations, methodological simplifications do not invalidate FEA per se; rather, they delineate the interpretative boundaries within which numerical findings should be considered. When applied comparatively, evaluating different restorative materials, cavity designs, or adhesive strategies under identical modeling assumptions, FEA remains a robust and internally consistent tool for identifying relative mechanical trends [125]. Transparent reporting of model assumptions, validation strategies, and sensitivity analyses is therefore essential to ensure scientific rigor and reproducibility.

In summary, the methodological validity of FEA models in adhesive restorative direct and indirect dentistry depends on a careful balance between computational feasibility and biomechanical realism. While current simulations inevitably rely on simplifying assumptions, ongoing advances in imaging, material characterization, and numerical techniques continue to enhance their fidelity. Critical appraisal of modeling choices remains essential to avoid overinterpretation and to ensure that FEA serves as a meaningful complement to experimental and clinical research in contemporary adhesive restorative dentistry. Importantly, recent methodological refinements in dental FEA increasingly reflect broader advancements in adhesive restorative dentistry and computational biomechanics. High-resolution micro-CT-based geometric reconstruction, improved characterization of contemporary resin-based materials, and the incorporation of more sophisticated interfacial modeling strategies indicate a progressive alignment between numerical simulation frameworks and the evolving clinical and material landscape of adhesive dentistry.

### 4.3. Clinical Relevance and Translational Value of FEA Findings

Finite element analysis provides clinically meaningful insight in adhesive restorative dentistry by enabling a mechanistic interpretation of how restorative design, material selection, and adhesive strategies influence stress distribution within the tooth–restoration complex. Although FEA does not directly predict clinical longevity, its primary clinical value lies in clarifying the biomechanical rationale underlying restoration performance and failure patterns, thereby supporting evidence-informed decision-making in daily practice [14,15,126].

From a clinical perspective, FEA consistently identifies stress-prone regions that are difficult or impossible to investigate experimentally, such as internal line angles, cervical enamel margins, and adhesive interfaces [26,27,127]. These numerical observations help explain clinically observed failure modes, including marginal degradation, interfacial debonding, and crack initiation in both direct and indirect resin-based restorations [88,89,90,91,92,128]. By allowing standardized comparisons between restorative strategies under controlled conditions, FEA supports rational choices regarding cavity design, restoration thickness, cuspal coverage, and material stiffness, particularly in structurally compromised posterior teeth [129,130].

For instance, FEA studies on extensive class II and MOD restorations consistently demonstrate that loss of marginal ridges significantly increases cuspal deflection and tensile stress concentration at cervical enamel margins when compared with more conservative class I cavity designs [41,42,43,44]. These findings provide a biomechanical rationale for limiting extensive direct restorations in structurally compromised posterior teeth and for considering partial cuspal coverage strategies when residual wall thickness is reduced. Similarly, numerical investigations evaluating the interaction between restorative material properties and cavity geometry show that elastic modulus mismatch between resin-based materials and dental tissues significantly influences stress transfer to enamel, dentin, and adhesive interfaces [34,36,49,50]. In indirect adhesive restorations, finite element simulations further indicate that restoration thickness and cement layer representation play a decisive role in stress localization at enamel margins and bonded interfaces [83,84,85,86,100,101,102,103,104].

In posterior restorations, numerical evidence provides a clear biomechanical explanation for the transition from intracoronal direct restorations to partial cuspal coverage strategies when marginal ridges and cuspal support are compromised. Finite element simulations consistently demonstrate that onlays, overlays, and tabletop restorations reduce cuspal deflection and redistribute tensile stresses more favorably than extensive intracoronal restorations, thereby supporting minimally invasive alternatives to full-coverage crowns [83,84,85,86,88,89,90,92,95]. These findings are particularly relevant in the context of adhesion-driven restorative principles, where structural preservation and stress modulation are prioritized over macro-retentive preparation designs.

Although less extensively represented in the literature, FEA investigations in anterior teeth highlight distinct biomechanical challenges related to non-axial loading and tensile stress concentration at incisal edges and adhesive interfaces [26,27]. In class III and IV restorations, restoration design, marginal configuration, and interfacial integrity appear to exert a greater influence on stress distribution than bulk material properties alone. Similarly, in class V cervical restorations, numerical simulations consistently demonstrate stress amplification driven by tooth flexure and elastic mismatch between restorative materials and dental substrates, providing a mechanical explanation for the susceptibility of these restorations to marginal degradation [69,70,71,76].

A further clinically relevant contribution of FEA lies in elucidating the central biomechanical role of adhesive interfaces. Models explicitly incorporating adhesive layers and resin cements demonstrate that variations in cement thickness, elastic modulus, and bonding assumptions substantially influence stress transfer between restoration and tooth structure [18,131]. These findings reinforce the concept that adhesion is not merely a procedural step, but a structural determinant of load distribution within the restored tooth.

Beyond theoretical stress mapping, several consistent trends emerging from FEA investigations can be translated into clinically actionable principles. Preservation of marginal ridges and residual cusp thickness consistently emerges as a dominant determinant of stress amplification, reinforcing the biomechanical rationale for conservative cavity design whenever clinically feasible [7,8,41,42,43,44]. In structurally compromised posterior teeth, cuspal coverage achieved through indirect adhesive restorations systematically reduces cuspal deflection and redistributes tensile stresses away from vulnerable cervical enamel regions, supporting partial coverage strategies as a minimally invasive alternative to full crowns [83,84,85,86,88,89,90,92,95]. Moreover, mechanical compatibility between restorative material, adhesive layer, and dental substrate appears to exert greater influence on stress distribution than absolute material stiffness alone [10,34,36,40]. Excessively rigid materials may increase interfacial stress concentration, whereas more compliant systems may better accommodate deformation at bonded interfaces. Numerical simulations also demonstrate that cement thickness and adhesive layer representation significantly affect stress localization patterns, underscoring the clinical importance of controlled luting procedures and meticulous adhesive protocol execution [10,100,101,102,103,104]. When interpreted collectively, these findings indicate that adhesive strategy should be regarded as a structural variable capable of modulating biomechanical performance rather than as a purely operative step.

At the same time, clinical interpretation of FEA findings must be contextualized within the time-dependent behavior of contemporary restorative materials. Conventional FEA models assume mechanical stability and chemical inertness, whereas experimental evidence indicates that several polymer-based restorative materials exhibit bioactive behavior, including ion release and physicochemical modifications under intraoral conditions [132,133,134]. Such processes may influence interfacial stability, stress transfer, and mechanical compatibility over time. Consequently, FEA findings should be interpreted as indicators of initial biomechanical performance and integrated with experimental data on material aging, ion release, and degradation phenomena when informing restorative material selection [132,133,134].

Importantly, acknowledging these limitations does not diminish the clinical value of FEA. Rather, it clarifies its appropriate role within a broader investigative framework. When integrated with laboratory experimentation and clinical evidence, FEA remains a powerful adjunct for understanding mechanical behavior at restoration placement and for guiding rational restorative decision-making within a minimally invasive and adhesion-driven paradigm.

### 4.4. Limitations and Future Perspectives of FEA in Adhesive Restorative Dentistry

Despite its recognized value as a biomechanical investigative tool, finite element analysis (FEA) presents inherent limitations that must be explicitly acknowledged to avoid overinterpretation of numerical findings in adhesive restorative dentistry. Many of these limitations arise from the need to balance computational feasibility with biomechanical realism, a compromise that inevitably affects model assumptions, outcome interpretation, and clinical translatability [30,135].

One of the primary constraints of FEA lies in the simplification of material behavior. Dental hard tissues, resin-based composites, adhesive systems, and resin cements are commonly modeled as homogeneous, isotropic, and linearly elastic materials, despite extensive evidence of anisotropy, viscoelasticity, and time-dependent mechanical behavior [29,51,72,95]. While such assumptions are generally acceptable for comparative analyses under controlled loading conditions, they limit the ability of numerical models to reproduce clinically relevant phenomena such as fatigue damage accumulation, stress relaxation, creep, and progressive interfacial degradation [39,40].

Future developments in materials science may enable the integration of experimentally derived time-dependent constitutive models into FEA frameworks, incorporating viscoelastic response, fatigue-dependent degradation, hygroscopic expansion, and ion-release–related chemo-mechanical interactions observed in contemporary bioactive restorative materials [132,133,134]. The inclusion of these parameters would allow simulations to move beyond initial stress distribution analysis toward a more realistic representation of long-term intraoral behavior.

A further major limitation concerns the representation of adhesive interfaces. Most dental FEA studies assume perfectly bonded conditions between enamel, dentin, adhesive layers, and restorative resin-based materials, thereby neglecting the presence of defects, incomplete hybrid layer formation, and chemical degradation that may occur clinically [31,36,136]. Even when adhesive layers are explicitly modeled, the complex chemo-mechanical interactions occurring at bonded interfaces remain difficult to reproduce numerically. As a result, FEA outcomes should be interpreted as representations of idealized or initial biomechanical compatibility rather than as predictors of long-term adhesive durability.

Advances in high-resolution imaging technologies, including micro-computed tomography, cone-beam computed tomography, and contemporary digital intraoral scanning systems, may significantly enhance the anatomical fidelity of finite element models. The incorporation of patient-specific geometries, more accurate cavity configurations, and realistic cement thickness distributions could reduce geometric idealization and improve the clinical relevance of stress predictions.

Loading and boundary conditions represent an additional source of uncertainty. Static axial loading remains frequently adopted in dental FEA, despite clear evidence that masticatory function involves multidirectional, cyclic, and time-dependent forces [137]. Simplified loading scenarios may underestimate stress concentrations in cervical regions, thin restorations, and adhesive interfaces, particularly in minimally invasive restorations and anterior teeth [69,70,71,77,78]. Similarly, the omission of thermo-mechanical effects limits the ability of current models to replicate the clinical environment, where temperature fluctuations and moisture interact with mechanical loading.

An important conceptual limitation of current FEA approaches in adhesive dentistry is the assumption that polymeric restorative materials remain mechanically stable and chemically inert over time. Experimental evidence demonstrates that several contemporary restorative materials exhibit bioactive behavior, including the release of fluoride, calcium, phosphate, hydroxyl, silicon, and strontium ions under intraoral-like conditions, leading to physicochemical changes such as water sorption, hygroscopic expansion, pH modulation, and potential alterations in elastic modulus [132,133,134,138]. These time-dependent processes are not captured by conventional FEA models, which therefore predominantly describe short-term mechanical behavior rather than the evolving performance of restorations in vivo.

When viewed in a broader context, the limitations of dental FEA become particularly evident when compared with applications of the finite element method in other dental specialties and in engineering disciplines. In implant dentistry, orthodontics, and endodontics, FEA has demonstrated higher predictive consistency owing to more standardized geometries, better-defined material properties, and clearer boundary conditions, allowing closer correlation between numerical outcomes and clinical behavior [139,140,141,142,143,144,145,146,147,148,149,150,151,152]. Beyond dentistry, FEA is extensively and successfully employed in biomedical and engineering fields such as cardiovascular biomechanics, orthopedic implant design, maxillofacial surgery, and fluid–structure interaction modeling, where material properties and failure criteria are often well characterized and experimentally validated [146,147,151,152,153,154]. These fields illustrate how the predictive power of FEA increases as model assumptions more closely approximate real-world conditions.

Artificial intelligence (AI) and machine learning methodologies may further expand the translational potential of FEA in adhesive restorative dentistry. In engineering and biomedical applications, AI has been used to optimize mesh generation, automate parameter calibration, and develop surrogate predictive models capable of approximating complex simulations with reduced computational cost. In the dental context, similar data-driven frameworks could support sensitivity analysis, probabilistic modeling of restoration performance, and integration of large experimental datasets, such as fatigue testing results or ion-release kinetics, into simulation workflows, thereby strengthening validation procedures and predictive robustness. Beyond conventional finite element formulations, emerging computational strategies such as meshless or particle-based methods have been explored in broader computational biomechanics. However, their application in adhesive restorative dentistry remains limited, and current evidence is still predominantly based on classical finite element frameworks.

Future developments in FEA are expected to progressively enhance its translational relevance in adhesive restorative dentistry. Advances in imaging technologies, including micro-computed tomography and high-resolution digital scanning, will further improve geometric fidelity and enable patient-specific modeling. Moreover, the incorporation of time-dependent material properties, fatigue behavior, and chemo-mechanical coupling into numerical simulations represents a promising avenue for capturing the evolving behavior of adhesive restorations. Integrating experimental data on ion release, aging, and mechanical degradation into FEA frameworks may allow future models to better approximate long-term clinical conditions and biological interactions.

Within this evolving landscape, FEA should be regarded not as a standalone predictive instrument, but as a component of a multidisciplinary investigative strategy that integrates numerical modeling with laboratory experimentation and clinical research. When used within its methodological boundaries and interpreted alongside complementary evidence, FEA remains a valuable tool for advancing biomechanical understanding and supporting the rational design of minimally invasive adhesion-driven restorative procedures.

## 5. Conclusions

This narrative review critically examined the application of finite element analysis (FEA) in polymer-based adhesive restorative dentistry, with particular emphasis on direct and indirect restorations across different cavity configurations and tooth types. By synthesizing the available literature, the review highlighted recurring biomechanical trends related to cavity design, cuspal support, material stiffness, adhesive interface modeling, and loading conditions. The analysis underscored that while FEA provides valuable mechanistic insights into stress distribution and structural behavior, its clinical interpretability remains strongly dependent on modeling assumptions, material characterization, and boundary condition definition. Simplified geometric representations, homogeneous material modeling, and idealized bonding conditions limit predictive accuracy but do not invalidate the comparative and interpretative role of FEA.

When integrated with experimental validation and clinical evidence, FEA represents a powerful adjunct tool for understanding biomechanical patterns, refining adhesive strategies, and supporting rational restorative planning. Continued methodological refinement and alignment with evolving material technologies and computational approaches are expected to further enhance its translational relevance.

## Figures and Tables

**Table 1:** Clinically oriented synthesis of finite element analysis findings in adhesive restorative dentistry, summarizing FEA-derived biomechanical insights, intrinsic methodological limitations, and clinical implications across direct and indirect resin-based adhesive restorations according to cavity class and tooth region.

Restorative Scenario	FEA-Derived Mechanical Insights	Intrinsic Methodological Limitations of FEA	Clinical Interpretation and Implications
**Posterior teeth—Class I direct restorations**	Stress concentrations mainly localized at enamel margins and adhesive interfaces; limited cuspal deflection when cavity geometry is conservative	Polymerization shrinkage and interfacial behavior often idealized; time-dependent stress relaxation not reproduced	Supports mechanical adequacy of conservative direct adhesive restorations when adhesion is properly established
**Posterior teeth—Class II direct restorations (single marginal ridge loss)**	Increased tensile stresses at cervical enamel and proximal box margins under occlusal loading	Static loading conditions may underestimate fatigue-related damage	Highlights the importance of cavity geometry optimization and adhesive integrity to maintain marginal stability
**Posterior teeth—Class II MOD direct restorations**	Pronounced cuspal deflection and stress amplification at internal line angles, cervical enamel, and adhesive interfaces	Linear elastic material models do not predict crack initiation or long-term failure	Provides biomechanical rationale for limiting extensive direct restorations and considering cuspal coverage strategies
**Anterior teeth—Class III direct restorations**	Shrinkage-induced tensile stresses concentrated at enamel cavosurface margins; limited global tooth deformation	Combined functional, parafunctional, and fatigue loading rarely simulated	Emphasizes the dominant role of marginal design and enamel bonding over bulk material stiffness
**Anterior teeth—Class IV direct restorations**	High tensile and shear stresses at the incisal edge and adhesive interface under oblique loading	Simplified loading protocols may not reproduce complex anterior contact dynamics	Supports restoration design optimization to mitigate stress concentration in incisal restorations
**Cervical region—Class V direct restorations**	Stress concentration at the cervical margin driven by tooth flexure and elastic mismatch between materials	Thermo-mechanical coupling and viscoelastic effects often simplified	Explains susceptibility to marginal degradation and guides material selection toward elastic compatibility
**Posterior teeth—Indirect intracoronal restorations (inlays)**	Partial recovery of tooth stiffness with persistent stress concentration at internal line angles and adhesive interfaces	Resin cement behavior and polymerization shrinkage frequently idealized	Suggests limited biomechanical benefit in severely weakened teeth
**Posterior teeth—Indirect cuspal coverage restorations (onlays/overlays)**	Reduced cuspal deflection and more favorable stress redistribution compared with direct restorations	Stress transfer remains sensitive to restoration thickness and adhesive layer assumptions	Supports minimally invasive cuspal coverage as an alternative to full-coverage crowns
**Ultra-conservative indirect restorations (tabletops/occlusal veneers)**	High stress localization at the enamel–cement interface, especially under non-axial loading	Thin restoration geometry amplifies sensitivity to modeling assumptions	Reinforces the central biomechanical role of enamel bonding and cement selection

## Data Availability

No new data were created or analyzed in this study.

## References

[1] Breschi L., Maravic T., Mazzitelli C., Josic U., Mancuso E., Cadenaro M., Pfeifer C.S., Mazzoni A. (2025). The Evolution of Adhesive Dentistry: From Etch-and-Rinse to Universal Bonding Systems. Dent. Mater..

[2] Pfeifer C.S., Lucena F.S., Tsuzuki F.M. (2025). Preservation Strategies for Interfacial Integrity in Restorative Dentistry: A Non-Comprehensive Literature Review. J. Funct. Biomater..

[3] Perdigão J., Araujo E., Ramos R.Q., Gomes G., Pizzolotto L. (2021). Adhesive Dentistry: Current Concepts and Clinical Considerations. J. Esthet. Restor. Dent..

[4] Ivanova S., Tomova Z., Vlahova A., Stoeva I.L., Vasileva E., Uzunova Y., Urumova M., Tomov D., Chonin A. (2026). Contemporary Use of Polymers in Dentistry: A Narrative Review. Polymers.

[5] Opdam N.J., van de Sande F.H., Bronkhorst E., Cenci M.S., Bottenberg P., Pallesen U., Gaengler P., Lindberg A., Huysmans M.C., van Dijken J.W. (2014). Longevity of Posterior Composite Restorations: A Systematic Review and Meta-Analysis. J. Dent. Res..

[6] Heintze S.D., Loguercio A.D., Hanzen T.A., Reis A., Rousson V. (2022). Clinical Efficacy of Resin-Based Direct Posterior Restorations and Glass-Ionomer Restorations—An Updated Meta-Analysis of Clinical Outcome Parameters. Dent. Mater..

[7] Neto M.A., Roseiro L., Messias A., Falacho R.I., Palma P.J., Amaro A.M. (2021). Influence of Cavity Geometry on the Fracture Strength of Dental Restorations: Finite Element Study. Appl. Sci..

[8] Forster A., Braunitzer G., Tóth M., Szabó B.P., Fráter M. (2019). In Vitro Fracture Resistance of Adhesively Restored Molar Teeth with Different MOD Cavity Dimensions. J. Prosthodont..

[9] Versluis A., Tantbirojn D., Pintado M.R., DeLong R., Douglas W.H. (2004). Residual Shrinkage Stress Distributions in Molars after Composite Restoration. Dent. Mater..

[10] Ausiello P., Apicella A., Davidson C.L. (2002). Effect of Adhesive Layer Properties on Stress Distribution in Composite Restorations—A 3D Finite Element Analysis. Dent. Mater..

[11] Magne P., Knezevic A. (2009). Simulated Fatigue Resistance of Composite Resin versus Porcelain CAD/CAM Overlay Restorations on Endodontically Treated Molars. Quintessence Int..

[12] Mario D., Mario A., Allegra C., Andrea B., Giuseppe T., Milena C., Annalisa M., Lorenzo B., Lorenzo L.M., Nicola S. (2022). The Influence of Indirect Bonded Restorations on Clinical Prognosis of Endodontically Treated Teeth: A Systematic Review and Meta-Analysis. Dent. Mater..

[13] Peskersoy C., Acar G. (2025). In Vitro Evaluation of the Mechanical Properties of Posterior Adhesive Restorations Fabricated Using Three Different Techniques. Polymers.

[14] Torres C.R.G., Mailart M.C., Pinatti R.F.A., da Silva D.F., Moreira J.C., Pereira T.C., Ruano V., de Holanda M.A.R., Barros P.C.A., Campos R.P. (2025). Clinical Performance of Restorations in Anterior Teeth Using Composites with Two Levels of Translucency: Split-Mouth Randomized Clinical Trial. J. Dent..

[15] Raedel M., Hertel S., Priess H.W., Mikeli A., Kopzon V., Bohm S., Walter M.H. (2024). Four-Year Outcomes of Class III and IV Anterior Restorations Based on a Subset of German Health Insurance Data. J. Adhes. Dent..

[16] Saleh S.A., Ebaya M.M., Ali A.I. (2025). Marginal and Internal Adaptation of Different Flowable Composite Restorations in Class V Cavities after Thermomechanical Cyclic Loading: In Vitro Study. BMC Oral Health.

[17] Incekara M.S., Karadas M. (2025). Clinical Comparison of Direct and Indirect Class II Composite Restorations: A Prospective 12-Month Follow-Up Study. BMC Oral Health.

[18] Bourgi R., Kharouf N., Cuevas-Suárez C.E., Lukomska-Szymanska M., Haikel Y., Hardan L. (2024). A Literature Review of Adhesive Systems in Dentistry: Key Components and Their Clinical Applications. Appl. Sci..

[19] Ausiello P., Gloria A., Maietta S., Watts D.C., Martorelli M. (2020). Stress Distributions for Hybrid Composite Endodontic Post Designs with and without a Ferrule: FEA Study. Polymers.

[20] Crins L.A.M.J., Opdam N.J.M., Huysmans M.C.D.N.J.M., Zhang Y., Loomans B.A.C. (2024). An In Vitro Evaluation of the Fatigue Behavior of Resin Composite Materials as Part of a Translational Research Cycle. Dent. Mater..

[21] Hennig C.L., Stöcker A., Nitzsche A., Marquetand J., Jacobs C., Jahn F. (2023). Influence of Root Post Materials and Aging on Fracture Strength and Marginal Gap Quality of Ceramic Crowns—An In Vitro Study. Materials.

[22] Di Francesco P., Bechir A., Popescu A.I., Chivu M.V., Dobrescu A.M., Comăneanu R.M., Târcolea M. (2025). Finite Element Analysis (FEA) of the Stress Behavior of Some Dental Materials. J. Med. Life.

[23] Ausiello P., Ciaramella S., De Benedictis A., Lanzotti A., Tribst J.P.M., Watts D.C. (2021). The Use of Different Adhesive Filling Material and Mass Combinations to Restore Class II Cavities under Loading and Shrinkage Effects: A 3D-FEA. Comput. Methods Biomech. Biomed. Eng..

[24] Correia A.M.O., Pereira V.E.M., Bresciani E., Platt J.A., Borges A.L.S., Caneppele T.M.F. (2019). Influence of Cavosurface Angle on the Stress Concentration and Gaps Formation in Class V Resin Composite Restorations. J. Mech. Behav. Biomed. Mater..

[25] Kintopp C.C.A., Diógenes A.N., Lopes R.T., Weber K.R., Rezende C.E.E., Kaizer M.D.R., Gonzaga C.C. (2025). Effect of Different Preparations and Restorative Materials on Partial Posterior Restorations: A 3D FEA Study Using μCT Data. J. Prosthet. Dent..

[26] Xu H., Jiang Z., Xiao X., Fu J., Su Q. (2012). Influence of Cavity Design on the Biomechanics of Direct Composite Resin Restorations in Class IV Preparations. Eur. J. Oral Sci..

[27] Grassi E.D.A., de Andrade G.S., de Carvalho A.B.G., Gasparro R., Mariniello M., Aliberti A., Ausiello P., Borges A.L.S. (2025). Evaluation of Internal and Marginal Shrinkage Stress in Adhesive Class III Cavities Restored with Different Resin Composite Combinations—A 3D-FEA Study. Dent. J..

[28] Karakaya K., Erdem R.Z. (2025). Stress Distribution in the Closure of Anterior Maxillary Diastemas Using Different Restorative Approaches: A Finite Element Analysis. Clin. Oral Investig..

[29] Fidancioğlu Y.D., Alkurt Kaplan S., Mohammadi R., Gönder H.Y. (2025). Three-Dimensional Finite Element Analysis (FEM) of Tooth Stress: The Impact of Cavity Design and Restorative Materials. Appl. Sci..

[30] Trivedi S. (2014). Finite Element Analysis: A Boon to Dentistry. J. Oral Biol. Craniofac. Res..

[31] Mokeem L.S., Garcia I.M., Melo M.A. (2023). Degradation and Failure Phenomena at the Dentin Bonding Interface. Biomedicines.

[32] Lee H.H., Majd H., Orrego S., Majd B., Romberg E., Mutluay M.M., Arola D. (2014). Degradation in the Fatigue Strength of Dentin by Cutting, Etching and Adhesive Bonding. Dent. Mater..

[33] Ouldyerou A., Mehboob H., Mehboob A., Merdji A., Aminallah L., Mukdadi O.M., Barsoum I., Junaedi H. (2023). Biomechanical Performance of Resin Composite on Dental Tissue Restoration: A Finite Element Analysis. PLoS ONE.

[34] Rodrigues F.P., Silikas N., Watts D.C., Ballester R.Y. (2012). Finite Element Analysis of Bonded Model Class I ‘Restorations’ after Shrinkage. Dent. Mater..

[35] Boaro L.C., Gonçalves F., Guimarães T.C., Ferracane J.L., Versluis A., Braga R.R. (2010). Polymerization Stress, Shrinkage and Elastic Modulus of Current Low-Shrinkage Restorative Composites. Dent. Mater..

[36] Ausiello P., Ciaramella S., Di Rienzo A., Lanzotti A., Ventre M., Watts D.C. (2019). Adhesive Class I Restorations in Sound Molar Teeth Incorporating Combined Resin-Composite and Glass Ionomer Materials: CAD-FE Modeling and Analysis. Dent. Mater..

[37] Tuncdemir M.T., Yeşilyurt N.G., Arıkan M. (2021). Comparison of the Stress Distribution in Class I and Class II Amalgam and Bulk-Fill Composite Restorations Using CAD-FEM Modeling. Int. J. Periodontics Restor. Dent..

[38] Mitthra S., Rajkumar K., Mahalaxmi S. (2017). Evaluation of Polymerization Shrinkage, Polymerization Shrinkage Stress, Wear Resistance, and Compressive Strength of a Silorane-Based Composite: A Finite Element Analysis Study. Indian J. Dent. Res..

[39] Ausiello P., Dal Piva A.M.O., di Lauro A.E., Garcia-Godoy F., Testarelli L., Tribst J.P.M. (2022). Mechanical Behavior of Alkasite Posterior Restorations in Comparison to Polymeric Materials: A 3D-FEA Study. Polymers.

[40] di Lauro A.E., Ciaramella S., Tribst J.P.M., Aliberti A., Ausiello P. (2024). Comparison of Bulk Polymeric Resin Composite and Hybrid Glass Ionomer Cement in Adhesive Class I Dental Restorations: A 3D Finite Element Analysis. Polymers.

[41] Ausiello P., Ciaramella S., Garcia-Godoy F., Martorelli M., Sorrentino R., Gloria A. (2017). Stress Distribution of Bulk-Fill Resin Composite in Class II Restorations. Am. J. Dent..

[42] Valin Rivera J.L., Gonçalves E., Vinicius Soares P., Milito G., Ricardo Perez J.O., Palacios Roque G.F., Valin Fernández M., Figueredo Losada H., Araújo Pereira F., Garcia del Pino G. (2022). The Restored Premolars Biomechanical Behavior: FEM and Experimental Moiré Analyses. Appl. Sci..

[43] Daher R., Feilzer A.J., Krejci I. (2016). Novel Non-Invasive Reinforcement of MOD Cavities on Endodontically Treated Teeth. J. Dent..

[44] Țuculină M.J., Staicu A.N., Munteanu M.C., Cumpătă C.N., Dimitriu B., Rîcă A.M., Beznă M.C., Popa D.L., Popescu A.D., Țîrcă T. (2023). Study on the Restoration of Class II Carious Cavities by Virtual Methods: Simulation of Mechanical Behavior. J. Funct. Biomater..

[45] Manchorova-Veleva N.A. (2011). Three-Dimensional Analysis of Cavity Wall Deformation after Composite Restoration of Masticatory Teeth. Folia Med..

[46] Valian A., Moravej-Salehi E., Geramy A., Faramarzi E. (2015). Effect of Extension and Type of Composite-Restored Class II Cavities on Biomechanical Properties of Teeth: A Three Dimensional Finite Element Analysis. J. Dent..

[47] Alp Ş., Gulec Alagoz L., Ulusoy N. (2020). Effect of Direct and Indirect Materials on Stress Distribution in Class II MOD Restorations: A 3D-Finite Element Analysis Study. Biomed. Res. Int..

[48] Srivastava B., Devi N.N., Gupta N., Singh R. (2018). Comparative Evaluation of Various Temperature Changes on Stress Distribution in Class II Mesial-Occlusal-Distal Preparation Restored with Different Restorative Materials: A Finite Element Analysis. Int. J. Clin. Pediatr. Dent..

[49] Kantardžić I., Vasiljević D., Lužanin O., Maravić T., Blažić L. (2018). Influence of the Restorative Procedure Factors on Stress Values in Premolar with MOD Cavity: A Finite Element Study. Med. Biol. Eng. Comput..

[50] Pereira R., Bicalho A.A., Franco S.D., Tantbirojn D., Versluis A., Soares C.J. (2016). Effect of Restorative Protocol on Cuspal Strain and Residual Stress in Endodontically Treated Molars. Oper. Dent..

[51] Gönder H.Y., Mohammadi R., Harmankaya A., Yüksel İ.B., Fidancıoğlu Y.D., Karabekiroğlu S. (2023). Teeth Restored with Bulk—Fill Composites and Conventional Resin Composites; Investigation of Stress Distribution and Fracture Lifespan on Enamel, Dentin, and Restorative Materials via Three-Dimensional Finite Element Analysis. Polymers.

[52] Sharma S., Ramesh S., Rayapudi J. (2023). Biomechanical Performance of Mandibular Molars with Deep Mesio-Occlusal-Distal Cavities Rehabilitated with Horizontal Posts: A 3D Finite Element Analysis. Int. J. Dent..

[53] Sengul F., Gurbuz T., Sengul S. (2014). Finite Element Analysis of Different Restorative Materials in Primary Teeth Restorations. Eur. J. Paediatr. Dent..

[54] Masoudi Nejad R., Ghahremani Moghadam D., Ramazani Moghadam M., Aslani M., Asghari Moghaddam H., Mir M. (2021). Fracture Behavior of Restored Teeth and Cavity Shape Optimization: Numerical and Experimental Investigation. J. Mech. Behav. Biomed. Mater..

[55] Li H., Yun X., Li J., Shi L., Fok A.S., Madden M.J., Labuz J.F. (2010). Strengthening of a Model Composite Restoration Using Shape Optimization: A Numerical and Experimental Study. Dent. Mater..

[56] Bansal P., Seth T., Kumar M., Bhatt M., Arora P., Gupta I., Chaudhary S., Akkanapally S., Arora A., Singh S. (2024). Comparative Evaluation of Stress Distribution and Deformation in Class II Cavities Restored with Two Different Biomimetic Restorative Materials: A Three-Dimensional Finite Element Analysis. Cureus.

[57] Xu J., Liang X., Hu L., Sun C., Zhang Z., Yang J., Wang J. (2025). How to Adaptively Balance ‘Classic’ or ‘Conservative’ Approaches in Tooth Defect Management: A 3D-Finite Element Analysis Study. BMC Oral Health.

[58] Thadathil Varghese J., Islam F., Farrar P., Prentice L., Prusty B.G. (2024). Multi-Response Optimisation Analysis of Material Properties in Dental Restorative Composites under the Influence of Thermal and Thermomechanical Stimuli—A 3D Finite Element Study. J. Mech. Behav. Biomed. Mater..

[59] Staicu A.N., Țuculină M.J., Cumpătă C.N., Rîcă A.M., Beznă M.C., Popa D.L., Popescu A.D., Diaconu O.A. (2024). A Finite Element Method Study on a Simulation of the Thermal Behaviour of Four Methods for the Restoration of Class II Cavities. J. Funct. Biomater..

[60] Boțilă M.-R., Popa D.L., Mercuț R., Iacov-Crăițoiu M.M., Scrieciu M., Popescu S.M., Mercuț V. (2024). A Finite Element Method Study of Stress Distribution in Dental Hard Tissues: Impact of Access Cavity Design and Restoration Material. Bioengineering.

[61] Magne P., Tan D.T. (2008). Incisor Compliance Following Operative Procedures: A Rapid 3-D Finite Element Analysis Using Micro-CT Data. J. Adhes. Dent..

[62] Magne P., Douglas W.H. (2000). Cumulative Effects of Successive Restorative Procedures on Anterior Crown Flexure: Intact Versus Veneered Incisors. Quintessence Int..

[63] Rathke A., Frehse H., Selinka A. (2025). Effect of Beveling Large Class II Cavities on the Enamel Marginal Quality of Direct Resin-Based Restorations. J. Clin. Med..

[64] Golsanamlou O., Estaki Z., Jamali Z. (2022). Bond Strength of Conventional Versus Modified Methods for Class IV Restorations in Primary Incisors: An In Vitro Study. J. Dent. Res. Dent. Clin. Dent. Prospect..

[65] Wu H., Li C., Dong L., Li X., Li X., Huang W., Yu S., Zhang Q., Yan X., Yuan X. (2025). Biomechanical Analysis on Mandibular Anterior Teeth during Unilateral Mandibular Molar Protraction with Different Movement Patterns: A Finite Element Analysis. BMC Oral Health.

[66] Romero M.F., Haddock F.J., Freites A.G., Brackett W.W., Brackett M.G. (2016). Restorative Technique Selection in Class IV Direct Composite Restorations: A Simplified Method. Oper. Dent..

[67] Liu J., Zhang J., Liu W., Liang S. (2025). Combining a CAD-CAM Composite Resin Palatal Wall with a Direct Composite Resin Layering Technique for the Restoration of a Large Class IV Fracture: A Clinical Report. J. Prosthet. Dent..

[68] Comba A., Baldi A., Carossa M., Paolone G., Stura I., Migliaretti G., Scotti N. (2023). A Three-Step Etch-and-Rinse vs a Universal Adhesive in Nanohybrid Composite Anterior Restorations: A Retrospective Clinical Evaluation. J. Adhes. Dent..

[69] Rees J.S. (2002). The Effect of Variation in Occlusal Loading on the Development of Abfraction Lesions: A Finite Element Study. J. Oral Rehabil..

[70] Soares P.V., Santos-Filho P.C., Soares C.J., Faria V.L., Naves M.F., Michael J.A., Kaidonis J.A., Ranjitkar S., Townsend G.C. (2013). Non-Carious Cervical Lesions: Influence of Morphology and Load Type on Biomechanical Behaviour of Maxillary Incisors. Aust. Dent. J..

[71] Ichim I., Schmidlin P.R., Kieser J.A., Swain M.V. (2007). Mechanical Evaluation of Cervical Glass-Ionomer Restorations: 3D Finite Element Study. J. Dent..

[72] Yaman S.D., Sahin M., Aydin C. (2003). Finite Element Analysis of Strength Characteristics of Various Resin Based Restorative Materials in Class V Cavities. J. Oral Rehabil..

[73] Eliguzeloglu E., Eraslan O., Omurlu H., Eskitascioglu G., Belli S. (2011). The Effect of Cavity Shape and Hybrid Layer on the Stress Distribution of Cervical Composite Restorations. Eur. J. Dent..

[74] Florescu A., Manea S., Hancu V., Manu R., Biclesanu C.F. (2017). The Influence of Cervical Cavity Shape on the Restoration Material Retention. A Finite Element Method Study. Mater. Plast..

[75] Guler M.S., Guler C., Cakici F., Cakici E.B., Sen S. (2016). Finite Element Analysis of Thermal Stress Distribution in Different Restorative Materials Used in Class V Cavities. Niger. J. Clin. Pract..

[76] Pereira F.A., Zeola L.F., de Almeida Milito G., Reis B.R., Pereira R.D., Soares P.V. (2016). Restorative Material and Loading Type Influence on the Biomechanical Behavior of Wedge Shaped Cervical Lesions. Clin. Oral Investig..

[77] Anhesini B.H., Landmayer K., Nahsan F.P.S., Pereira J.C., Honório H.M., Francisconi-Dos-Rios L.F. (2019). Composite vs. Ionomer vs. Mixed Restoration of Wedge-Shaped Dental Cervical Lesions: Marginal Quality Relative to Eccentric Occlusal Loading. J. Mech. Behav. Biomed. Mater..

[78] Ichim I.P., Schmidlin P.R., Li Q., Kieser J.A., Swain M.V. (2007). Restoration of Non-Carious Cervical Lesions Part II. Restorative Material Selection to Minimise Fracture. Dent. Mater..

[79] Pai S., Bhat V., Patil V., Naik N., Awasthi S., Nayak N. (2020). Numerical Three-Dimensional Finite Element Modeling of Cavity Shape and Optimal Material Selection by Analysis of Stress Distribution on Class V Cavities of Mandibular Premolars. J. Int. Soc. Prev. Community Dent..

[80] Rees J.S., Jacobsen P.H. (1998). The Effect of Cuspal Flexure on a Buccal Class V Restoration: A Finite Element Study. J. Dent..

[81] Kampanas N.S., Antoniadou M. (2018). Glass Ionomer Cements for the Restoration of Non-Carious Cervical Lesions: Biomechanical Considerations. J. Funct. Biomater..

[82] Pai S., Naik N., Patil V., Kaur J., Awasti S., Nayak N. (2019). Evaluation and Comparison of Stress Distribution in Restored Cervical Lesions of Mandibular Premolars: Three-Dimensional Finite Element Analysis. J. Int. Soc. Prev. Community Dent..

[83] Dejak B., Mlotkowski A. (2008). Three-Dimensional Finite Element Analysis of Strength and Adhesion of Composite Resin versus Ceramic Inlays in Molars. J. Prosthet. Dent..

[84] Dejak B., Młotkowski A. (2020). A Comparison of MvM Stress of Inlays, Onlays and Endocrowns Made from Various Materials and Their Bonding with Molars in a Computer Simulation of Mastication—FEA. Dent. Mater..

[85] Jiang W., Bo H., Yongchun G., LongXing N. (2010). Stress Distribution in Molars Restored with Inlays or Onlays with or without Endodontic Treatment: A Three-Dimensional Finite Element Analysis. J. Prosthet. Dent..

[86] Rocca G.T., Krejci I. (2007). Bonded Indirect Restorations for Posterior Teeth: From Cavity Preparation to Provisionalization. Quintessence Int..

[87] Lin C.L., Chang Y.H., Liu P.R. (2008). Multi-Factorial Analysis of a Cusp-Replacing Adhesive Premolar Restoration: A Finite Element Study. J. Dent..

[88] Ausiello P., Rengo S., Davidson C.L., Watts D.C. (2004). Stress Distributions in Adhesively Cemented Ceramic and Resin-Composite Class II Inlay Restorations: A 3D-FEA Study. Dent. Mater..

[89] Ausiello P., Ciaramella S., Garcia-Godoy F., Gloria A., Lanzotti A., Maietta S., Martorelli M. (2017). The Effects of Cavity-Margin-Angles and Bolus Stiffness on the Mechanical Behavior of Indirect Resin Composite Class II Restorations. Dent. Mater..

[90] Gomes de Carvalho A.B., de Andrade G.S., Mendes Tribst J.P., Grassi E.D.A., Ausiello P., Saavedra G.S.F.A., Bressane A., Marques de Melo R., Borges A.L.S. (2021). Mechanical Behavior of Different Restorative Materials and Onlay Preparation Designs in Endodontically Treated Molars. Materials.

[91] Yang H., Park C., Shin J.H., Yun K.D., Lim H.P., Park S.W., Chung H. (2018). Stress Distribution in Premolars Restored with Inlays or Onlays: 3D Finite Element Analysis. J. Adv. Prosthodont..

[92] Nikolova N., Raykovska M., Petkov N., Tsvetkov M., Georgiev I., Koytchev E., Iankov R., Dimova-Gabrovska M., Gusiyska A. (2025). The Integration of Micro-CT Imaging and Finite Element Simulations for Modelling Tooth-Inlay Systems for Mechanical Stress Analysis: A Preliminary Study. J. Funct. Biomater..

[93] Ausiello P., Ciaramella S., Fabianelli A., Gloria A., Martorelli M., Lanzotti A., Watts D.C. (2017). Mechanical Behavior of Bulk Direct Composite versus Block Composite and Lithium Disilicate Indirect Class II Restorations by CAD-FEM Modeling. Dent. Mater..

[94] Yamamoto T., Takeishi S., Momoi Y. (2007). Finite Element Stress Analysis of Indirect Restorations Prepared in Cavity Bases. Dent. Mater. J..

[95] Yamanel K., Caglar A., Gülsahi K., Ozden U.A. (2009). Effects of Different Ceramic and Composite Materials on Stress Distribution in Inlay and Onlay Cavities: 3-D Finite Element Analysis. Dent. Mater. J..

[96] Durand L.B., Guimarães J.C., Monteiro Junior S., Baratieri L.N. (2015). Effect of Ceramic Thickness and Composite Bases on Stress Distribution of Inlays—A Finite Element Analysis. Braz. Dent. J..

[97] Sellan P.L.B., Campaner L.M., Tribst J.P.M., Dal Piva A.M.O., de Andrade G.S., Borges A.L.S., Bresciani E., Lanzotti A., Ausiello P. (2021). Functional or Nonfunctional Cusps Preservation for Molars Restored with Indirect Composite or Glass-Ceramic Onlays: 3D FEA Study. Polymers.

[98] Jung M.K., Jeon M.J., Kim J.H., Son S.A., Park J.K., Seo D.G. (2024). Comparison of the Stress Distribution in Base Materials and Thicknesses in Composite Resin Restorations. Heliyon.

[99] Dejak B., Młotkowski A. (2015). A Comparison of Stresses in Molar Teeth Restored with Inlays and Direct Restorations, Including Polymerization Shrinkage of Composite Resin and Tooth Loading during Mastication. Dent. Mater..

[100] Al Sunbul H., Silikas N., Watts D.C. (2016). Polymerization Shrinkage Kinetics and Shrinkage-Stress in Dental Resin-Composites. Dent. Mater..

[101] Borges A.L.S., Dal Piva A.M.d.O., Moecke S.E., de Morais R.C., Tribst J.P.M. (2021). Polymerization Shrinkage, Hygroscopic Expansion, Elastic Modulus and Degree of Conversion of Different Composites for Dental Application. J. Compos. Sci..

[102] Kaisarly D., Gezawi M.E. (2016). Polymerization Shrinkage Assessment of Dental Resin Composites: A Literature Review. Odontology.

[103] Cornacchia T.M., Silva G.C., Magalhaes C.S., Moreira A.N., Las Casas E.B. (2014). Analysis of Stresses during the Polymerization Shrinkage of Self-Curing Resin Cement in Indirect Restorations: A Finite-Element Study. Indian J. Dent. Res..

[104] Campaner L.M., Alves Pinto A.B., Demachkia A.M., Paes-Junior T.J.d.A., Pagani C., Borges A.L.S. (2021). Influence of Cement Thickness on the Polymerization Shrinkage Stress of Adhesively Cemented Composite Inlays: Photoelastic and Finite Element Analysis. Oral.

[105] Chen Y.C., Lin C.L., Hou C.H. (2021). Investigating Inlay Designs of Class II Cavity with Deep Margin Elevation Using Finite Element Method. BMC Oral Health.

[106] Wicaksono S., Prasetia W., Muryani A., Dirgantara T., Mahyuddin A.I. (2023). Finite Element Stress Analysis of Dental Cement Application on Endocrown and Onlay Restoration. Aust. Endod. J..

[107] Pable G., Saha S.G., Saha M.K., Agarwal R.S., Meena S.S., Poddar G. (2025). Comparative Evaluation of Stress Distribution in Maxillary Premolar Restored with Onlay Fabricated with Different Restorative Materials—A Three-Dimensional Finite Element Analysis Study. J. Conserv. Dent. Endod..

[108] Tribst J.P.M., Dal Piva A.M.O., Penteado M.M., Borges A.L.S., Bottino M.A. (2018). Influence of Ceramic Material, Thickness of Restoration and Cement Layer on Stress Distribution of Occlusal Veneers. Braz. Oral Res..

[109] Chen Q., Luo S., Wang Y., Chen Z., Li Y., Meng M., Li Y., Xiao N., Dong Q. (2024). Three-Dimensional Finite Element Analysis of Occlusal Stress on Maxillary First Molars with Different Marginal Morphologies Restored with Occlusal Veneers. BMC Oral Health.

[110] Mei M.L., Chen Y.M., Li H., Chu C.H. (2016). Influence of the Indirect Restoration Design on the Fracture Resistance: A Finite Element Study. Biomed. Eng. Online.

[111] Baldi A., Scattina A., Comba A., Peroni L., Rossi T., Scotti N. (2026). Effects of Different Cement-Restorative Material Combinations in Full-Coverage Onlay Restorations: A FEA Study. Dent. Mater..

[112] Babaei B., Shouha P., Birman V., Farrar P., Prentice L., Prusty G. (2022). The Effect of Dental Restoration Geometry and Material Properties on Biomechanical Behaviour of a Treated Molar Tooth: A 3D Finite Element Analysis. J. Mech. Behav. Biomed. Mater..

[113] Weimann D., Morgenthal A., Schwendicke F., Fleck C., Razi H. (2021). Substantial Regional Differences in the Biomechanical Behavior of Molar Treated with Selective Caries Tissue Removal Technique: A Finite Element Study. Dent. Mater..

[114] Fouquet V., Larsen N., Stchepinsky A.C., Vennat E., Benoit A., Tapie L. (2024). A Parametrical Finite Element Analysis for Functionally Graded Material Overlay Restoration. J. Mech. Behav. Biomed. Mater..

[115] Oliveira A.A., Ribeiro M.L.P., Costa P.V.M., Pereira R.D., Versluis A., Veríssimo C. (2022). The Effect of Filling Technique on the Cuspal Strain, Polymerization Shrinkage Stress, Enamel Crack Formation and Depth of Cure of Restored Molars. Dent. Mater..

[116] Choi A.H., Conway R.C., Ben-Nissan B. (2014). Finite-Element Modeling and Analysis in Nanomedicine and Dentistry. Nanomedicine.

[117] Viceconti M., Olsen S., Nolte L.P., Burton K. (2005). Extracting Clinically Relevant Data from Finite Element Simulations. Clin. Biomech..

[118] Lin C.L., Chang W.J., Lin Y.S., Chang Y.H., Lin Y.F. (2009). Evaluation of the Relative Contributions of Multi-Factors in an Adhesive MOD Restoration Using FEA and the Taguchi Method. Dent. Mater..

[119] Moda M.D., Fagundes T.C., Briso A.L.F., Dos Santos P.H. (2018). Analysis of the Bond Interface between Self-Adhesive Resin Cement to Eroded Dentin In Vitro. PLoS ONE.

[120] Li Z., Du J., Yi H., Mao Z., Du K., Li T., Hao P., Wang B. (2025). Machine Learning Framework for Evaluating Microscale Interface Cohesive Zone Models of Polymer Composites. ACS Appl. Mater. Interfaces.

[121] Kozák V., Vala J., Derevianko A. (2025). Modelling Structural Material Damage Using the Cohesive Zone Approach Under Operational Conditions. Materials.

[122] Wciślik W., Pała T. (2021). Selected Aspects of Cohesive Zone Modeling in Fracture Mechanics. Metals.

[123] Maqableh A.M., Hatamleh M.M. (2023). Cohesive Zone Modeling of Pull-Out Test for Dental Fiber-Silicone Polymer. Polymers.

[124] Hashemipour M.A., Mohammadpour A., Nassab S.A. (2010). Transient Thermal and Stress Analysis of Maxillary Second Premolar Tooth Using an Exact Three-Dimensional Model. Indian J. Dent. Res..

[125] Jiang J., Sun J., Ma H., Wang J., Huang Z., Zhou S. (2023). Stress Intensity Factor of a Cracked Molar Restored with Different Materials and Designs: A 3D-FEA. J. Mech. Behav. Biomed. Mater..

[126] Fernández E., Gil A.C., Caviedes R., Díaz L., Bersezio C. (2025). Clinical Longevity of Direct Dental Restorations: An Umbrella Review of Systematic Reviews. J. Esthet. Restor. Dent..

[127] Magne P. (2007). Efficient 3D Finite Element Analysis of Dental Restorative Procedures Using Micro-CT Data. Dent. Mater..

[128] Liu T., Huang Y., Li Y., Meng J., Liu Y., Wei Y., Huang Y., Zhou Q., Yang W., Yan F. (2025). Effect of Different Restorative Design and Materials on Stress Distribution in Cracked Teeth: A Finite Element Analysis Study. BMC Oral Health.

[129] Mohammadi R., Alkurt Kaplan S., Harmankaya A., Gönder H.Y. (2025). Investigation of Stress Distribution and Fatigue Performance in Restored Teeth Using Different Thicknesses of Adhesive Materials and Different Restorative Materials: 3D Finite Element Analysis (FEM). Materials.

[130] Eraslan Ö., Eraslan O., Eskitaşcıoğlu G., Belli S. (2011). Conservative Restoration of Severely Damaged Endodontically Treated Premolar Teeth: A FEM Study. Clin. Oral Investig..

[131] Yasin Gönder H., Mohammadi R., Harmankaya A., Burak Yüksel İ., Seda Gültekin D. (2022). Investigation of the Effects of Adhesive Materials of Different Types and Thicknesses on Dental Tissue Stress via FEM Analysis. Biomed. Res. Int..

[132] Aliberti A., Di Duca F., Triassi M., Montuori P., Scippa S., Piscopo M., Ausiello P. (2025). The Effect of Different pH and Temperature Values on Ca^2+^, F^−^, PO_4_^3−^, OH^−^, Si, and Sr^2+^ Release from Different Bioactive Restorative Dental Materials: An In Vitro Study. Polymers.

[133] Aliberti A., Gasparro R., Triassi M., Piscopo M., Ausiello P., Tribst J.P.M. (2025). Fluoride Release from Pediatric Dental Restorative Materials: A Laboratory Investigation. Dent. J..

[134] Aliberti A., Garcia-Godoy F., Borges A.L.S., Tribst J.P.M., Gasparro R., Mariniello M., Ausiello P. (2025). Calcium, Phosphate and Fluoride Ionic Release from Dental Restorative Materials for Elderly Population: An In Vitro Analysis. Front. Oral Health.

[135] Maravić T., Comba A., Mazzitelli C., Bartoletti L., Balla I., di Pietro E., Josic U., Generali L., Vasiljević D., Blažić L. (2022). Finite Element and In Vitro Study on Biomechanical Behavior of Endodontically Treated Premolars Restored with Direct or Indirect Composite Restorations. Sci. Rep..

[136] Zhang Z., Zheng K., Li E., Li W., Li Q., Swain M.V. (2016). Mechanical Benefits of Conservative Restoration for Dental Fissure Caries. J. Mech. Behav. Biomed. Mater..

[137] Moga R.-A., Olteanu C.D., Delean A.G. (2024). The Importance of Boundary Conditions and Failure Criterion in Finite Element Analysis Accuracy---A Comparative Assessment of Periodontal Ligament Biomechanical Behavior. Appl. Sci..

[138] Di Lauro A., Di Duca F., Montuori P., Dal Piva A.M.O., Tribst J.P.M., Borges A.L.S., Ausiello P. (2023). Fluoride and Calcium Release from Alkasite and Glass Ionomer Restorative Dental Materials: In Vitro Study. J. Funct. Biomater..

[139] Gasparro R., Renno F., De Vita S., Lanzotti A., Martorelli M., Penta F., Sammartino G., Ausiello P. (2025). Loading Pressure Induced by 4 mm Implants on the Inferior Alveolar Nerve: A 3D Finite Element Analysis Model. J. Clin. Med..

[140] Ausiello P., Tribst J.P.M., Ventre M., Salvati E., di Lauro A.E., Martorelli M., Lanzotti A., Watts D.C. (2021). The Role of Cortical Zone Level and Prosthetic Platform Angle in Dental Implant Mechanical Response: A 3D Finite Element Analysis. Dent. Mater..

[141] Falcinelli C., Valente F., Vasta M., Traini T. (2023). Finite Element Analysis in Implant Dentistry: State of the Art and Future Directions. Dent. Mater..

[142] Abdelhafeez M.M. (2024). Applications of Finite Element Analysis in Endodontics: A Systematic Review and Meta-Analysis. J. Pharm. Bioallied Sci..

[143] Komaki H., Chae J.M., Park J.H., Hamanaka R., Yoshida N. (2025). Biomechanical Analysis of Mandibular Molar Intrusion with or without an Archwire Constriction Bend: A Finite Element Study. Am. J. Orthod. Dentofac. Orthop..

[144] Durmus M., Bulut M., Hezenci Y. (2025). A Comparative 3D Finite Element Analysis of Mandibular Advancement with Clear Aligners and Twin Block Appliances. BMC Oral Health.

[145] Yamaguchi-Higuchi R., Hamanaka R., Komaki H., Emori T., Tominaga J.Y., Horiguchi Y., Iwata S., Ogawa K., Kitaura A., Yoshida N. (2025). Biomechanical Effect of Combined Use of Mini-Screws and Aligners in Preventing Uncontrolled Tipping of the Incisor and Mesial Tipping of the Molar: A Three-Dimensional Finite Element Study. Prog. Orthod..

[146] Taylor C.A., Hughes T.J., Zarins C.K. (1998). Finite Element Modeling of Three-Dimensional Pulsatile Flow in the Abdominal Aorta: Relevance to Atherosclerosis. Ann. Biomed. Eng..

[147] Throop A., Sudbury N., Timmins L.H., Baradaran H., Weiss J.A., Arzani A. (2026). Comparative Analysis of Open-Source Finite Element Method Solvers for Computational Fluid Dynamics Performance in a Carotid Artery Model. J. Biomech. Eng..

[148] Muryani A., Prasetia W., Aripin D., Dharsono H.D.A., Rajion Z.A., Wicaksono S. (2026). Impact of Various Sleeve Materials on Temperature Variations During Guided Endodontic Access Cavity Preparation Utilizing Finite-Element Analysis. Clin. Exp. Dent. Res..

[149] Karatas Telli G., Samur Erguven S., Kilinc Y., Atac M.S., Sencimen M. (2026). Comparison of the Biomechanical Behavior of Different Fixation Configurations Following Le Fort I Advancement and Inferior Repositioning Surgery: A Three-Dimensional Finite Element Analysis. J. Craniomaxillofac. Surg..

[150] Li Y., Liu J., Zhang B., Zhang F., Tian Z., Zhang J. (2025). Biomechanical Analysis of Femoral Stress Response during Squatting: A Combined Multibody Dynamics and Finite Element Approach. J. Orthop..

[151] Guo Z., Song Y., Li X. (2026). Decoupling Analysis of Ultrasonic Scattering Characteristics in Porous Polycrystalline Materials Using Phase Field and Finite Element Methods. Ultrasonics.

[152] Shash Y.H., Elden R.H. (2025). Integration of Finite Element Method and Artificial Intelligence for Evaluating PEEK Composites in Rib Cage Reconstruction Process under Impact Conditions. J. Mater. Sci. Mater. Med..

[153] Shash Y.H., Elden R.H. (2025). Computational Analysis of L4-L5 Interspinous Process Devices and Interbody Fusion Spacers Using Ceramic and Polymeric Materials via Finite Element Modeling and Artificial Intelligence. Sci. Rep..

[154] Cui Y., An B., Wu Z., Shang Z., Zhang X. (2025). Research on Shape-Performance Integrated Monitoring Technology for Planetary Gearboxes Based on the Integration of Artificial Intelligence, Finite Element Analysis, and Multibody Dynamics Simulation. Sensors.

